# Neural patterns of word processing differ in children with dyslexia and isolated spelling deficit

**DOI:** 10.1007/s00429-021-02255-2

**Published:** 2021-03-23

**Authors:** Agnieszka Dębska, Chiara Banfi, Katarzyna Chyl, Gabriela Dzięgiel-Fivet, Agnieszka Kacprzak, Magdalena Łuniewska, Joanna Plewko, Anna Grabowska, Karin Landerl, Katarzyna Jednoróg

**Affiliations:** 1 Laboratory of Language Neurobiology, Nencki Institute of Experimental Biology, Polish Academy of Sciences, Warsaw, Poland; 2 Institute of Psychology, University of Graz, Graz, Austria; 3 Faculty of Psychology, Warsaw University, Warsaw, Poland; 4 Faculty of Psychology, SWPS University of Social Sciences and Humanities, Warsaw, Poland; 5 BioTechMed-Graz, Graz, Austria; 6 Department of Cognitive Science, Macquarie University, Sydney, NSW Australia

**Keywords:** Reading, Spelling, Speech–print convergence, Dyslexia, Isolated spelling deficit, FMRI

## Abstract

There is an ongoing debate concerning the extent to which deficits in reading and spelling share cognitive components and whether they rely, in a similar fashion, on sublexical and lexical pathways of word processing. The present study investigates whether the neural substrates of word processing differ in children with various patterns of reading and spelling deficits. Using functional magnetic resonance imaging, we compared written and auditory processing in three groups of 9–13-year olds (*N* = 104): (1) with age-adequate reading and spelling skills; (2) with reading and spelling deficits (i.e., dyslexia); (3) with isolated spelling deficits but without reading deficits. In visual word processing, both deficit groups showed hypoactivations in the posterior superior temporal cortex compared to typical readers and spellers. Only children with dyslexia exhibited hypoactivations in the ventral occipito-temporal cortex compared to the two groups of typical readers. This is the result of an atypical pattern of higher activity in the occipito-temporal cortex for non-linguistic visual stimuli than for words, indicating lower selectivity. The print–speech convergence was reduced in the two deficit groups. Impairments in lexico-orthographic regions in a reading-based task were associated primarily with reading deficits, whereas alterations in the sublexical word processing route could be considered common for both reading and spelling deficits. These findings highlight the partly distinct alterations of the language network related to reading and spelling deficits.

**Supplementary Information:**

The online version contains supplementary material available at 10.1007/s00429-021-02255-2.

## Introduction

A central question in written language research is the extent to which the cognitive and neural systems underlying reading and spelling are independent or overlapping (Tainturier and Rapp 2001; Jones and Rawson 2016). According to the *shared-components* view, reading and spelling rely on the same phonological and orthographic components. However, the dissociation between reading and spelling deficits suggests underlying differences. According to the *distinct-components* view, the sublexical and lexical paths of word processing differ for spelling and reading processes. Frith (1980, 1985) proposed that spelling deficits are associated with degraded orthographic representations that are sufficient for reading but not for more demanding operations like orthographic decisions and spelling. Since spelling requires more knowledge of grapheme-to-phoneme associations, it is easier for most people to read a word than to spell it accurately (Holmes and Carruthers 1998).

Previous neuroimaging studies have shown alterations of the sublexical and lexical routes of word processing associated with combined reading and spelling deficits (Richlan et al. 2009, 2011; Paulesu et al. 2014; Grigorenko 2001; McCandliss and Noble 2003; Pugh et al. 2000; Sandak et al. 2004; Shaywitz and Shaywitz 2005). However, because of the strong relationship between reading skills and spelling (correlation from 0.4 to 0.8, e.g., Tierney and Shanahan 1991; Berninger et al. 2002; Dębska et al. 2019), it is unknown which effects of atypical neural organization are related to deficits in one skill but not the other.

Evidence for a dissociation between underlying components of reading and spelling skills comes from research on isolated and combined reading and spelling deficits (Kemény et al. 2018; Gangl et al. 2018; Moll and Landerl 2009; Banfi et al. 2019). Children with isolated spelling deficit (ISD) have a selective orthographic deficit but, compared to children with dyslexia, age-adequate reading skills (American Psychiatric Association 2013). Dissociations between reading and spelling deficits can be observed in the general population (Bogdanowicz 2011; Fayol et al. 2009; Moll and Landerl 2009; Moll et al. 2014), with slightly different prevalence rates across countries due to different national diagnostic criteria, different tests, and orthographic transparency.

Previous studies have shown that children with ISD exhibit altered activity of structures belonging to a sublexical stream of word processing (Borkowska et al. 2014; Banfi et al. 2019) and difficulties in phonological awareness (Wimmer and Mayringer 2002; Döhla et al. 2018; Torppa et al. 2017). Results on lexico-orthographic processing are more contradictory. The network responsible for effective lexico-orthographic processing includes the left ventral occipito-temporal cortex (vOT, Lerma-Usabiaga et al. 2018; Dehaene et al. 2002). This network encompasses a particular region within the left fusiform gyrus: Visual Word Form Area (VWFA, Yeatman et al. 2013). It is known to be specifically related to processing of written word-forms (Cohen et al. 2002). VWFA is considered as storage for orthographic representations (Lerma-Usabiaga et al. 2018; Dehaene et al. 2002). Decreased activity in the left vOT was reported in a sample of 11 German-speaking ISD participants who engaged in an orthographic decision task (Gebauer et al. 2012). A more recent fMRI study involving children reading out loud partly confirmed these findings (Banfi et al. 2020), as decreased functional activity was observed in ISD at the whole-brain level in the left fusiform gyrus. Note, however, that this cluster was more anterior and medial than the “classic” location of the VWFA (Vogel et al. 2012). In a ROI analysis, targeting a vOT cluster corresponding to the location of the VWFA, Banfi et al. (2020) found no evidence for divergent reading-related functional activity in children with ISD as compared to typically developing children. Outside of fMRI studies, behavioral and ERPs studies have also shown no straightforward evidence in favor of divergent lexico-orthographic processing between typically developing and ISD children (Gangl et al. 2018; Kemény et al. 2018). This is different from dyslexia, where an orthographic processing deficit is evident for reading and spelling. On the neural level, we thus expect to observe differences between dyslexia (that show severe reading deficit) and ISD in word processing in the vOT.

During typical development of the lexico-orthographic path, the selectivity of the VWFA to written words emerges in response to orthographic stimuli along with reduced activation in other visual, non-linguistic stimuli (Maurer et al. 2007, 2011; Brem et al. 2013; Centanni et al. 2017, 2018). Existing cross-sectional studies on growing selectivity to print showed that children aged 5–14 have letter sensitivity similar to adults, but underspecified selectivity (Centanni et al. 2017; Blackburne et al. 2014). Centanni et al. (2018) also showed that greater letter sensitivity, but not selectivity in the left VWFA, is related to reading scores. In contrast, one longitudinal study (Centanni et al. 2019) showed that children before the reading onset, who subsequently developed dyslexia in the second grade, showed reduced responses to both print and novel false fonts in the left VWFA throughout. The lack of specialization for words in the VWFA in poor readers might come from weaker inhibition of non-preferred categories like false fonts (“selectionist view”, Cantlon et al. 2011; Brem et al. 2010), weaker response to real words but not false fonts (Kronschnabel et al. 2013), or decreased activation to both kinds of stimuli (Chyl et al. 2019a, b; Hervais-Adelman et al. 2019; Centanni et al. 2019).

The sublexical route development is associated with growing print–speech convergence in the temporoparietal path of word processing. The speech network becomes sensitive to print processing through reading acquisition (Monzalvo and Dehaene-Lambertz 2013). The level of overlapping print and speech activations in the left middle and superior temporal gyri (MTG/STG) positively correlates with the reading level in beginning readers (Chyl et al. 2018). Furthermore, the level of print–speech convergence in the left STG/MTG (Marks et al. 2019) and left inferior frontal gyrus (IFG) (Preston et al. 2016) predicts reading outcomes 1 or 2 years later. The individual level of phonological awareness (PA) has also been positively associated with the extent of overlap in activation for print and speech in the left pSTG (Frost et al. 2009). This observation opens an interesting discussion on how print–speech convergence is associated with reading acquisition on the one side, and growing reading-related skills, like phonological knowledge or spelling, on the other side.

In our study, we aimed to extend our understanding of the neural basis of reading vs. spelling deficits. We compared behavioral and neural responses to spoken and written words versus other non-linguistic stimuli in three groups of children: (1) a control group of typical readers and spellers; (2) readers with dyslexia, characterized by reading and spelling deficits; (3) children with ISD having reading skills comparable to the control group. We first considered functional patterns of word processing and then focused on two components of literacy acquisition: (1) The selectivity to written words in the VWFA, which reflects the quality of orthographic lexicon; (2) The print–speech convergence (Rueckl et al. 2015) in the sublexical, temporoparietal route of word processing.

First, we expected groups with phonological processing deficits (children with dyslexia and with ISD) to show the atypical organization of print-to-speech convergence in the left STG. Second, we expected to distinguish between competing views of the origins of the selectivity in VWFA. In a selectionist view, one would expect to observe stronger responses to non-linguistic stimuli vs. written words in dyslexia. In contrast, a reduction in functional activity related to visually presented stimuli would indicate a generalized down-regulation of visual processing. We also tested whether impaired selectivity in the VWFA is uniquely linked to reading deficits (the group with dyslexia), or whether it affects a group of typical readers with ISD.

## Methods

### Participants

Children (*N* = 104, 8–13 y.o.) were part of a larger cohort from the study on neural correlates of dyslexia conducted at the Nencki Institute. All participants had typical IQ (above 85), were right-handed, Polish-speaking, born at term (> 37 weeks), and had no history of neurological impairments or ADHD. Children included in the *control group of typical readers and spellers* (*N* = 42) scored at least at the 4th sten in two reading tests and in a spelling test (writing to dictation). On the normalized scale from 1st to 10th, results below the 4th sten correspond to the bottom 16th percentile in the normal distribution. Children assigned to the *dyslexia grou*p (*N* = 38) achieved low scores (below 16th percentile) in both reading tests. Children with *Isolated Spelling Deficit *(*ISD,* *N* = 24) were typical readers (scored above the cut-off point in both reading tasks), and achieved low scores (below 16th percentile) in the spelling task. We applied the double (fluency and accuracy) criteria of dyslexia, because in transparent orthography with consistent grapheme-to-phoneme mapping reading difficulties might be reflected in reading speed even when accuracy is at a relatively high level (Verhoeven and Keuning 2018). Children that scored below 4th sten in one reading task but above this cut-off point in the second reading tasks were treated as borderline cases and were not included in the final group. Nonverbal intelligence was measured with the Wechsler Intelligence Scale for Children – Revised (Polish adaptation: Matczak et al. 2008). The socioeconomic status assessment was based on The Barratt Simplified Measure of Social Status (BSMSS; Barratt 2006). Groups did not differ in age, sex, socioeconomic status, or nonverbal IQ. For demographic, reading, and spelling performance details, see Table 1. The study was approved by the ethical committee (University of Social Sciences and Humanities) and is in compliance with the Declaration of Helsinki. The data published in Dębska et al. 2019 included a partially overlapping group of participants, but the in-scanner task was different (in Dębska et al. 2019: auditory phonological task on pseudowords, here: visual and auditory word task).

**Table 1:** Participants’ characteristics

	Controls	Dyslexia	Isolated spelling deficit	
	*n* = 42	*n* = 38	*n* = 24	
Sex (girls / boys)	19 / 23	11 / 27	6 / 18	Chi^2^ = 3.6; *p* = 0.16
Age (years)	10.14 (0.88)	10.26 (1.0)	10.59 (1.1)	*F*(2,101) = 1.5; *p* = 0.223
Socioeconomic status	108 (15)	100 (22)	97 (23)	*F*(2,101) = 2.9; *p* = 0.07
WISC-R IQ	116 (12)	114 (12)	114 (11)	*F*(2,101) = .55; p = 0.57
Reading accuracy^1,2^	5.71 (0.94)	2.08 (0.67)	5.38 (1)	*F*(2,101) = 196; *p* < .001,*η*^2^ = 0.8 DYS < CON = ISD, *p* < 0.001
Reading speed^1,2^	5.38 (1.1)	2.32 (0.66)	5.2 (1.1)	*F*(2,101) = 112; *p* < 0.001, *η*^2^ = 0.7 DYS < CON = ISD, *p* < 0.001
Spelling to dictation^1,2^	5.29 (1.5)	2.3 (1.14)	2.71 (0.46)	*F*(2,101) = 67; *p* < 0.001,*η*^2^ = 0.6 CON > DYS = ISD, *p* < 0.001
Phonological awareness^1^	5.19 (1.5)	3.62 (2)	3.88 (1.5)	*F*(2,101) = 9; *p* < 0.001,*η*^2^ = 0.2 CON > DYS = ISD, *p* < 0.05
Rapid Automatized Naming^2^	51 (7)	63 (15)	50 (10)	*F*(2,101) = 14.74; *p* < 0.001,*η*^2^ = 0.2 DYS > CON = ISD, *p* < 0.001
Words reading per minute	83 (24)	45 (14)	79 (22)	*F*(2,101) = 30.79; *p* < .001,*η*^2^ = 0.4 DYS < CON = ISD, *p* < 0.001
Pseudowords reading per minute	45 (8)	28 (7)	45 (9)	*F*(2,101) = 53.65; *p* < 0.001,*η*^2^ = 0.5 DYS < CON = ISD, *p* < 0.001

For ANOVA analyses, values of F are reported for group effects

^1^Normalised sten score from 1 to 10

^2^Digits and letters: number of seconds needed to finish a trial, higher score represents lower performance

^3^Normalised tests used as an assessment criteria

### Behavioral measures

Children were tested with the Polish normalized battery for dyslexia diagnosis in two versions: one for 3rd and beginning 4th graders and one for late 4th and 5th graders (Bogdanowicz et al. 2008). The sight-word reading test measures the accuracy of reading, while the pseudoword reading test measures speed and fluency. The sight-word reading test for younger children consisted of 50 words to read and, for older children, 85 words. Words differed in the level of complexity and frequency of occurrence in Polish between the two versions. In pseudoword reading, the task was to accurately read a list of pseudowords in 60 s (max. 70 items). This test has identical sets of items in the two versions. The pseudowords were pronounceable but had no close word neighbors. The spelling test also had 2 versions, 1 for younger children (a story consisting of 85 words) and another for older children (a story consisting of 171 words). The individual results in all tasks for every child were transformed into normalized (sten) scores based on the psychometric scale from the battery. Aside from two reading and one spelling task from the battery of dyslexia diagnosis (Bogdanowicz et al. 2008), participants completed an independent reading task called the Decoding Test (Szczerbiński and Pelc-Pękala, 2013) which measured sight-word and pseudoword reading per minute. Phonological awareness tasks measured accuracy in pseudoword matching, syllable, and phoneme analysis and synthesis, as well as phonological memory (Bogdanowicz et al. 2008). Rapid automatized naming (RAN) was tested with subtests of letters and digits (Polish version: Fecenec et al. 2013). All behavioral data were analyzed with one-way ANOVA and post hoc corrected for multiple comparisons with the Bonferroni correction (see Table 1).

### fMRI task and procedure

fMRI data were acquired on a 3T Siemens Trio scanner using a whole-brain echo-planar imaging sequence with a 12-channel head coil (32 slices, slice-thickness 4 mm, TR = 2000 ms, TE = 30 ms, flip angle = 80°, FOV = 220 mm2, matrix size: 64 × 64, voxel size 3 × 3 × 4 mm). Anatomical data were acquired using a T1-weighted sequence (176 slices, slice-thickness 1 mm, TR = 2530 ms, TE = 3.32 ms, flip angle = 7°, matrix size: 256 × 256, voxel size 1 × 1 × 1 mm). First, children were familiarized with the experiment and MR environment in a mock-scanner. In the fMRI scanner, children were presented with print and speech circuit localizers with four conditions: (1) real printed words (print); (2) real spoken words (speech); (3) symbol strings; and (4) vocoded speech. Conditions (3) and (4) served as the non-linguistic control and were matched to the print and speech conditions in physical characteristics (length and visual complexity in the case of symbol strings and dynamic frequency and amplitude content in the case of vocoded speech). In condition (3), symbol strings contained the same words as in the printed word condition, but written with Wingdings font. Word–symbol contrasts were used in previous studies as indicating print specialization (Maurer et al. 2005, 2007, 2011). Printed and spoken words were highly frequent short Polish words selected from the Polish CHILDES database of child-directed speech (Haman et al. 2015). In conditions (1) and (2), high-frequency, one or two-syllable Polish words were used. The mean number of letters was 4.16 (SD = 0.86) for the print condition and 4.14 (SD = 0.85) for the speech condition. The mean number of phonemes was 3.85 (SD = 0.75) for the print as well as for the speech conditions. They were matched for lexical parameters such as the number of letters, phonemes and syllables, parts-of-speech, and frequency according to two different corpora (for details, see Supplementary Materials, S1, Chyl et al. 2018). In auditory control trials, the spoken words from condition (2) were vocoder processed with Praat (Boersma and Weenink 2001). This process divides the speech signal into three frequency bands, applies the dynamic amplitude contour of the original to a noise source, then recombines these into a unitary signal again. This results in an auditory stimulus that retains the same dynamic frequency and amplitude pattern of the original, but largely destroys phonetic content. Auditory stimuli were delivered through MRI-compatible noise cancelling headphones (CRS) at approximately 70 dB, and visual stimuli were presented on a computer screen (black letters/symbols on a white background).

The task contained two runs, each lasting 5 min 2 s. In total, there were 24 trials per condition presented pseudorandomly with 96 stimuli per condition. No condition was repeated more than three times in a row. In each trial, four different stimuli from the same condition were presented in a rapid periodic stimulation. Each visual stimulus was presented for 250 ms, followed by a 200 ms blank screen. Each auditory stimulus was presented for 800 ms. ‘Jittered’ intertrial intervals were used with occasional ‘null’ trials resulting in ITIs ranging from 4 to 13 s (6.25 s on average). Stimuli were presented using Presentation software (Neurobehavioral Systems, Albany, CA). Children were instructed to pay attention to the stimuli. The task was designed to elicit strong activations in the language network due to the rapid exposure to linguistic stimuli. This is an established method in fMRI (Malins et al. 2016; Chyl et al. 2018, 2019a, b) and EEG studies (Lochy et al. 2015) testing print sensitivity in children. Previous studies have shown that this task is responsive to differences in the reading level (Malins et al. 2016; Chyl et al. 2018, 2019a, b).

### fMRI data processing and analysis

The neuroimaging data preprocessing and analyses were performed using Statistical Parametric Mapping (SPM12, Welcome Trust Centre for Neuroimaging, London, UK). Images were realigned to the participant mean. Next, T1-weighted images were segmented using pediatric tissue probability maps (Template-O-Matic toolbox). The functional images were normalized to MNI space. Finally, the normalized images were smoothed with an 8 mm isotropic Gaussian kernel. The data were modeled for each run using the canonical hemodynamic response function convolved with the experimental conditions and fixation periods. In addition to adding movement regressors to the design matrix, the ART toolbox was used to reject motion-affected volumes by modeling them in the design matrix. Subjects were included if a minimum of 80% of volumes from each run were artifact-free. Artifactual volumes were identified using a movement threshold of 3 mm and a rotation threshold of 0.05 radians (based on Raschle et al. 2012). All children in the final fMRI group (*N* = 104) fulfilled the inclusion criteria. Children from Control, Dyslexia, and ISD groups did not differ significantly in the number of motion-affected volumes (*F*(2,101) = 2, *p* = 0.14, CON*mean* = 7, DYS*mean* = 11, ISD*mean* = 8). A random effect GLM was computed for each participant and condition. We conducted a subject-level analysis of the experimental conditions: print, speech, symbol strings, vocoded speech as well as rest, including motion parameters and separate regressors for each volume identified as motion-affected by ART toolbox. In the first-level analysis, contrasts were generated for each condition against rest (print > rest, speech > rest) and between conditions (print > symbol strings, speech > vocoded-speech).

### Whole-brain analysis

In the second level of analysis, one-way ANOVA with three groups was used to analyze the main effect of group in each contrast: print > rest, speech > rest, print > symbol strings, speech > vocoded-speech. Print > symbol strings and speech > vocoded speech contrasts were used to identify brain circuits specific for print and speech, respectively. Pairwise group comparisons were masked by the significant main effect activation. All results are reported with the significance threshold *p* < 0.005 uncorrected at voxel level, and *p* < 0.05 family-wise error at cluster level.

### Selectivity to written words in VWFA

To test the hypothesis of reduced selectivity to printed stimuli in VWFA in groups with dyslexia and ISD, we created the independent ROI based on the work of Lerma-Usabiaga et al. (2018) (middle occipital temporal sulcus, coordinates: − 42, − 58, − 10, sphere: 8 mm). The sphere was masked by active voxels identified from the whole-brain contrasts for print processing across all participants. We chose this ROI based on the analysis showing its proximity to the region classically appointed as responsible for orthographic processing (Dehaene et al. 2002; Gaillard et al. 2006) and its anatomical connection with the language-oriented temporoparietal cortex and the inferior frontal gyrus (Lerma-Usabiaga et al. 2018).

### Print–speech convergence analysis

To illustrate print–speech convergence regions on the whole-brain level we used a null (AND) conjunction between 1) print < rest, speech < rest contrasts and 2) print < symbol strings, speech < vocoded speech contrasts. To analyze statistical differences between overlapping print–speech activations, we calculated the number of voxels for each subject that were activated (*p* < 0.05) for print and speech (conjoint probability, *p* < 0.0025) in selected regions of interest (ROIs). As in Preston et al. (2016) for print and speech–rest contrasts, we included the total number of voxels activated in print < rest condition and speech < rest condition within the grey matter mask as a covariate and treated them as a relative, individual level of activation for each subject. In the case of print and speech minus control conditions, the individual level of activation was controlled by the overall activation in the control conditions (symbol strings and vocoded speech). To create ROIs, we used anatomical masks created with AAL atlas in the WFU_pickatlas toolbox. Based on previous literature concerning print–speech convergence (Marks et al. 2019; Preston et al. 2016; Chyl et al. 2019a, b), we chose four ROIs belonging to the reading network: the left and right IFG and the left and right STG/MTG. Next, all between-group differences in a number of activated voxels in ROIs were tested within one repeated measure ANOVA model.

## Results

### Behavioral results

The group with dyslexia underperformed in all tasks (see Table 1) compared to the control group. Groups with dyslexia and ISD showed a similar, low phonological awareness level, but the group with dyslexia scored lower than ISD in word and pseudoword reading per minute and RAN. The ISD group did not differ from controls in word and pseudoword reading tasks and the rapid naming task but performed significantly worse in the phonological awareness task. As evident from Table 1, the ISD group shows a similar spelling level as the dyslexia group and a similar reading level as the control group. The chi^2^ test comparing distribution of normalized sten scores within control and ISD groups was nonsignificant for both reading tests: accuracy (chi^2^(3) = 3.83, *p* = 0.28) and speed (chi^2^(4) = 4.96, *p* = 0.97).

### fMRI results

#### Whole-brain analysis

**Fig. 1:**
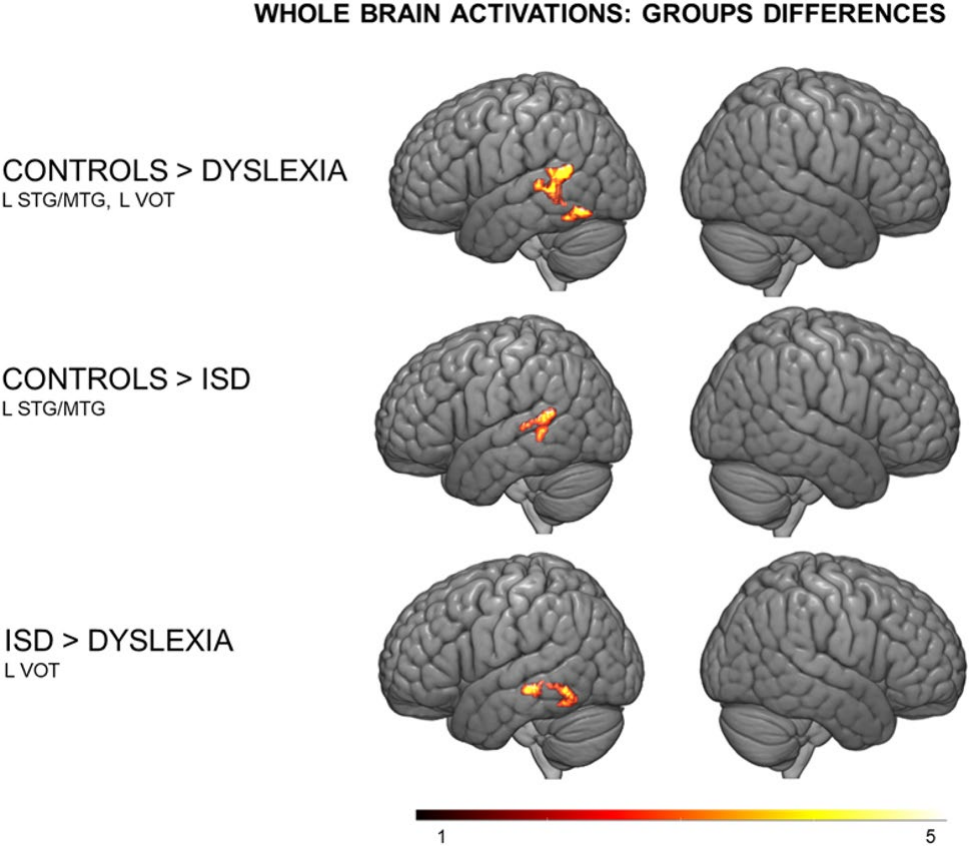
Whole-brain main ANOVA effects and post hoc in contrast print > symbol strings

The main effect of Group for the print > symbol strings condition was located in the left STG/MTG and the left ventral occipito-temporal cortex (− 58, − 50, 14, *F* = 10.66, *Z* = 3.83, pFWEcorr = 0.009). The threshold for a significant cluster (*p* = 0.05) within the whole brain mask was 736 voxels. Post hoc comparisons revealed that the groups with dyslexia and ISD underactivated the left STG/MTG cluster compared to controls. Both deficit groups showed hypoactivations in the posterior superior temporal cortex compared to typical readers and spellers. Still, only children with dyslexia exhibited hypoactivations in the ventral occipito-temporal cortex compared to the two groups of typical readers. There was no significant main effect of Group in any other contrasts: speech > vocoded speech, print > rest, speech > rest, symbols > rest, vocoded speech > rest at a chosen threshold (height threshold *p* < 0.005, FWEc, *p* < 0.05, see Fig. 1 and Table 2).

**Table 2:** Significant group effect across groups of children in print > symbol strings contrast (ISD: Children with isolated spelling deficit)

Brain region	H	*x*	*y*	*z*	*T*	*p*	Voxels
Controls > Dyslexia							
Inferior occipital and temporal, fusiform	L	− 46	− 60	− 14	3.98	< 0.05	144
Middle and superior temporal Gyri*	R	− 58	− 50	14	4.21	< 0.005	484
Controls > ISD							
Middle and superior temporal Gyri	L	− 56	− 48	14	3.56	< 0.05	162
ISD > Dyslexia							
Inferior temporal, fusiform	L	− 42	− 26	− 10	3.95	< 0.05	214

Group effect was tested with one-way ANOVA. Post hoc comparisons were tested with small volume correction in the region masked by the main group effect, height threshold *p* < 0.005, FWEc, *p* < 0.05

### Print–speech convergence analysis

#### Co-activations of print and speech versus control conditions

**Fig. 2:**
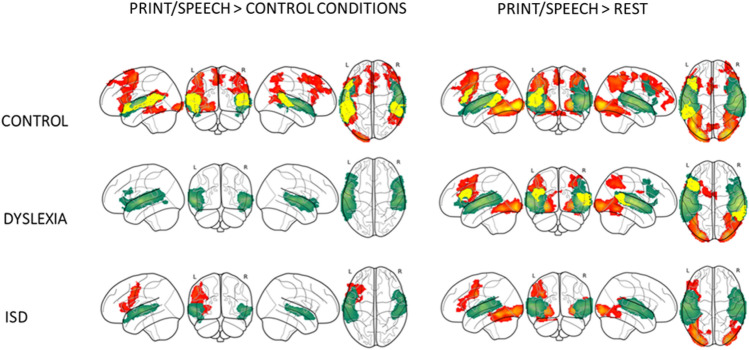
Response to printed (red) or spoken words (green) and conjunction (yellow) versus non-linguistic control stimuli or rest. One-sample t tests

According to the conjunction analysis (see Table S1 and Fig. 2), the control group showed print–speech convergence for print- and speech-specific contrasts in the bilateral STG/MTG and the left IFG. Dyslexia and ISD groups showed no regions with overlapping co-activations in either the print-specific or speech-specific level at the chosen threshold. Individual voxel convergence analysis within the ROIs (Left, Right: IFG; Left, Right: STG/MTG) tested with a repeated-measures ANOVA model revealed a significant group effect (*F*(2,102) = 43.96, *p* < 0.001, *η*^2^ = 0.3), ROI effect (*F* (3,303) = 28, *p* = 0.001, *η*^2^ = 0.2), and interaction effect (*F*(6,303) = 4.24, *p* = 0.001, *η*^2^ = 0.07). As for the significant Group effect, post hoc tests with the Bonferroni correction showed a larger number of overlapping voxel activations in controls than in the group with dyslexia at the trend level (*p* = 0.052). The ROI effect showed significantly more overlapping voxels in the L STG/MTG than in other ROIs (*p* < 0.001 for all comparisons) and the least in the R IFG (*p* < 0.01 for all comparisons). The interaction effect was due to a larger number of overlapping voxels in controls than in dyslexia in the L STG/MTG (dyslexia > controls, *p* = 0.002), and larger number of voxels in ISD than in dyslexia in the same region at a trend level (ISD > dyslexia, *p* = 0.062).

### Co-activations of print and speech versus rest

In this conjunction analysis, convergence occurred in the left STG/MTG as well as in the left IFG in the control group. Convergence occurred in the right STG and the left IFG for the group with dyslexia. ISD showed no regions with overlapping co-activations at the chosen threshold (see Table S2 and Fig. 2). However, individual voxel convergence analysis for ROIs in the repeated-measures ANOVA model revealed no significant main effects or interactions (Group effect (*F*(2,102) = 0.15, *p* < 0.85), ROI effect (*F*(3,303) = 0.007, *p* = 0.99), Interaction effect (*F*(6,303) = 0.63, *p* = 0.7)).

### Selectivity to written words in VWFA

**Fig. 3:**
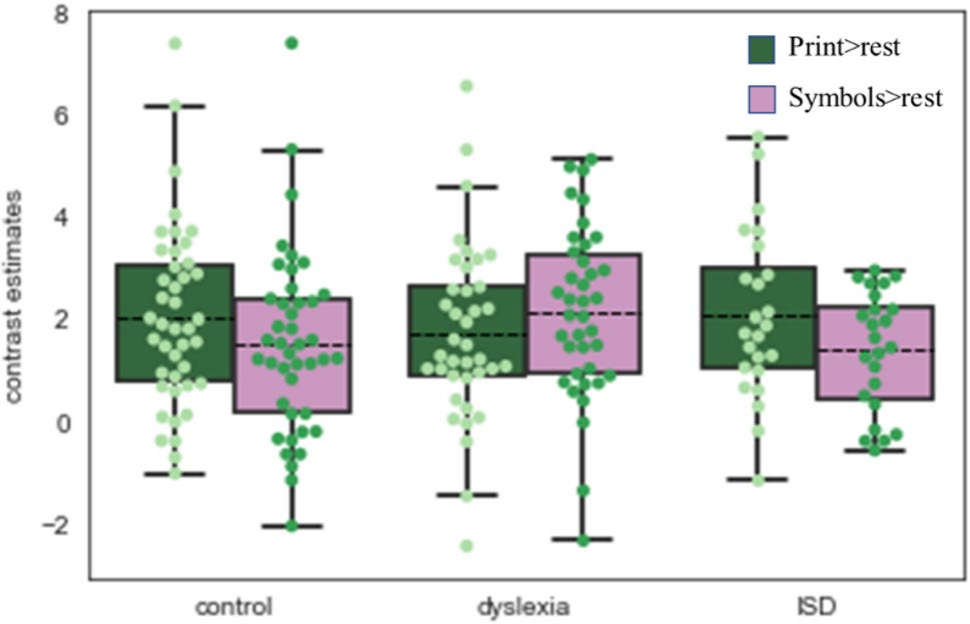
Average contrast estimates in print > rest (green) and symbols > rest (purple) contrasts in VWFA

Following the Kubota et al. (2019) line of reasoning, we compared responses to words and symbol strings separately to see if groups with dyslexia and/or ISD differ from typical readers and spellers in activation of words and symbol strings. We wanted to compare not only the differences between activity to print vs symbol strings but also the average activity to words and symbol strings among groups. Therefore, we conducted the repeated measure ANOVA model with a within-subject factor (Condition: symbol strings, print) and between-subject factor (Group: control, dyslexia, ISD). It revealed a significant interaction effect (*F*(2,102) = 6.6, *p* < 0.005, *η*^2^ = 0.12), whereas main effects of Condition and Group were not significant (Condition, *F*(1,102) = 3.63, *p* = 0.059, *η*^2^ = 0.035, Group, *F*(2,102) = 0.165, *p* = 0.848, *η*^2^ = 0.035). Post hoc tests with the Bonferroni correction revealed higher activity for words than symbol strings in controls (*p* = 0.014, *η*^2^ = 0.058) and ISD (*p* = 0.016, *η*^2^ = 0.056) groups (average contrast estimates for Print > Rest: ISD = 2.07, CON = 2.04, Symbols > Rest: ISD = 1.39, CON = 1.52). The group with dyslexia showed a trend for higher activity to symbol strings than print (average contrast estimates for Print > Rest: DYS = 1.72; Symbols > Rest: DYS = 2.15, *p* = 0.06, *η*^2^ = 0.03, see Fig. 3).

## Discussion

This study investigated the organization of the neural network for written and spoken word processing associated with selective and combined deficits in reading and spelling. Behaviorally, groups with dyslexia and ISD showed a similar, low level of phonological awareness compared to typical readers and spellers. This finding is in line with previous evidence in younger German-speaking children (Wimmer and Mayringer 2002). As put forward by Moll and Landerl (2009), phonological deficits in ISD might hamper the development of precise orthographic representations. Our results solidify the link between phonological deficit and spelling problems (see also Dębska et al. 2019).

When considering the whole group of participants, reading and spelling showed high intercorrelations (spelling–reading speed, *r* = 0.55, *p* < 0.001; spelling–reading accuracy, *r* = 0.061, *p* < 0.001). However, only spelling level but not reading level correlates with the socioeconomic status of children (spelling-SES, *r* = 0.28, *p* = 0.004, reading accuracy-SES, *r* = 0.027, *p* = 0.79, reading speed-SES, *r* = 0.083, *p* = 0.4, with significant difference between correlation coefficients *z* = 1.922, *p* = 0.027). SES is known to be a strong predictor of literacy (Noble and McCandliss 2005) and is associated with different reading-related skills including early print experience, the quality of schooling, and home literacy (Hecht et al. 2000). In our study, the spelling level shows greater sensitivity to differences in social environment than the reading level. This may be due to the age of the school children tested (from 3rd to 5th grade) which are more experienced with print and with Polish orthography which is more consistent in reading than in spelling.

When we considered neural patterns of word processing, typical readers and spellers showed higher activity for the print > symbol strings contrast in the left posterior STG/MTG cluster than both groups with reading and/or spelling deficits. The left posterior STG belongs to the dorsal stream of speech processing and is responsible for encoding phonological information (Hickok and Poeppel 2004; Price 2012). Children with phonological deficits show reduced activity in the STG compared to controls (e.g. McCandliss and Noble 2003). The left STG, MTG, and STS are assumed to constitute a hub, not only for speech sound processing, but also for establishing phonological representations of words (Gow 2012). A recent longitudinal fMRI study (Wang et al., 2019) showed that the level of activity in the pSTG at 6 years of age predicts reading development 2 years later. Overall, for children with dyslexia and ISD, lower activity in the pSTG when processing words might be a hallmark for difficulties in operating on phonological representations of written words on a sublexical level of processing. Such difficulties influence both their reading as well as spelling level.

Only children with dyslexia exhibited hypoactivations in the print > symbol strings contrast in the vOT compared to two groups of typical readers (ISD and controls). An atypical (reversed) pattern, with higher VWFA activity for non-linguistic visual stimuli vs written words, was observed in poor readers but not in the two groups of typical readers. In this respect, it is interesting to note that brain activity in a simple contrast such as words vs rest did not differentiate significantly between the groups of children. What differentiated poor and typical readers was not the general sensitivity to words in the VWFA, but rather altered selectivity to words vs other non-linguistic stimuli. This is in line with the selectionist view (Cantlon et al. 2011) and other studies showing lower selectivity in the VWFA for words in poor readers driven by the higher response to objects (Kubota et al. 2019) or false fonts (Pleisch et al. 2019). Similarly, Olulade et al. (2015) showed that children with dyslexia having an age similar to our sample (around 10 years old) lack word selectivity in the WVFA. Longitudinal studies on children with dyslexia (Centanni et al. 2019; Chyl et al. 2019a, b) comment on the generally lower response to all visual stimuli observed in this group. However, the general lower sensitivity may subsequently impede their selectivity for real words. We cannot exclude this possibility, since in our study, children were older and more experienced with written words and symbols than in many studies on print selectivity in the VWFA.

Interestingly, no atypical pattern of written word selectivity in the VWFA was found in the group of poor spellers (ISD). Since the VWFA is supposed to store orthographic representations (Lerma-Usabiaga et al. 2018l Dehaene et al. 2002) and participates in orthographic access for spelling *and* reading (Purcell et al. 2011, 2017; Rapp and Lipka 2011; Tsapkini and Rapp 2010), one might expect a degraded response to written words in the VWFA in children with a spelling deficit. Instead, in our study, children with ISD showed similar activity to written words compared to controls and a typical pattern of lesser engagement of non-linguistic stimuli in the VWFA. Previous eye-tracking and ERPs studies based on reading paradigms showed no severe impairment in ISD children with respect to orthographic processing (Kemény et al. 2018; Gangl et al. 2018), while deficient lexico-semantic access was reported (Kemény et al. 2018). In a recent fMRI study, Banfi et al. (2020) found no evidence of altered brain activity during a reading-aloud task in children with ISD as compared to typically developing children in dorsal and ventral regions of the reading network. This is in line with the *distinct-component* view. Although children with ISD showed a deficient *output* lexicon on the behavioral level, it seems that their *input* orthographic lexicon is intact or it is detailed enough for reading but not for spelling (Frith 1980, 1985). Alternatively, passive print processing tasks with short, high-frequency words are not sufficient to yield differences in VWFA activity between ISD and typical spellers. However, we showed that children with ISD are characterized by unaffected sensitivity and selectivity to print in the VWFA in a reading-based task.

Recently, Stevens et al. (2017) found functional connectivity between the VWFA defined at the individual level and the posterior portion of the STG corresponding to the area of Wernicke. Consistent with this, structural connections between a phonological STG region and the ventral occipito-temporal cortex were reported (Beer et al. 2013). VWFA was thus shown to be specifically involved in real word processing due to its functional and structural connectivity with a phonological area. The connectivity between phonological and orthographic regions is crucial for skilled reading. The decreased general connectivity in language networks was found to be impaired in dyslexic readers (Gonalzez et al. 2016; Koyama et al. 2013) suggesting that altered reading might be caused by the deficient connectivity rather than dysfunction in a specific brain area.

Taking that into account, it would be interesting to test whether connectivity between phonological and orthographic regions is indeed impaired in children with ISD. This might explain their difficulties in orthographic processing along with the phonological dysfunction.

The groups with dyslexia and ISD did not show significant overlapping activations for print and speech. This suggests that alterations in the organization of the speech network have occurred to accommodate print. Only the control group manifested consistent print–speech convergence in the bilateral STG/MTG and the left IFG. Likewise, in a recent study on younger (5–9 y.o.) Polish and American children using the same fMRI tasks, the bilateral IFG and STG/MTG regions showed overlapping print–speech activations for children in both languages (Chyl et al. 2019a, b). Similar print–speech convergence was reported in previous studies on typical beginning and skilled adult readers of different orthographies (Rueckl et al. 2015; Preston et al. 2016; Marks et al. 2019). The level of print–speech convergence in older children with typical development from the 3rd to 5th grades has never been investigated before. Our results confirm the stable, universal pattern of this cortical mechanism, visible after a few years of schooling. Also, in the individual voxel analysis, the left STG/MTG had a higher level of print–speech convergence in the control group compared to the group with dyslexia. Marks et al. (2019) and Preston et al. (2016) showed that the level of print–speech convergence in the language network in beginning readers predicts reading gains 1 or 2 years later. This suggests that ineffective reorganization may represent one of the factors explaining later reading deficit. Importantly, based on our cross-sectional results, we can say that alterations in print–speech convergence at different levels (sensitivity and selectivity) are not restricted to dyslexia but, to some extent, may also affect groups of typical, average readers with selective orthographic difficulties. This is the first study that has found noticeable alterations in print–speech convergence patterns in groups of children with reading and/or spelling deficits, both showing poor phonological skills.

Multiple cognitive deficits theory assumes that difficulties in reading/spelling might have sources in many different cognitive deficits (e.g. phonology (e.g. Ramus et al. 2013), visual attention (e.g. Valdois et al. 2004) or sensorimotor skills (e.g. Cornelissen et al. 1998). So far, we know that reading and spelling deficits share an underlying cognitive deficit in phonological awareness (e.g., Dohla and Heim 2016; Moll and Landerl 2009; Dębska et al. 2019). This finding corresponds to our results of lower activity in a phonological region (pSTG) in both dyslexia and ISD group during word processing compared to typical readers and spellers. Lately, Dohla et al. (2018) showed that auditory and visual magnocellular deficits, previously also implicated in dyslexia (Ramus et al. 2003) might be present in the spelling deficit. One study on neurocognitive subtypes of dyslexia (Jednoróg et al. 2014) showed grey matter volume differences between three dyslexia subtypes: with phonological awareness and magnocellular-dorsal skills, with impairments in rapid naming and auditory attention shifting and with a double deficit (phonological and rapid naming). However, further research on the neural basis of ISD subtypes are needed.

One of the study’s limitations is that, although children were explicitly instructed to carefully look at the screen and pay attention to the stimuli, we did not provide any explicit task, e.g. target detection, which would control for the participant's attentiveness. Thus, we cannot exclude the possibility that some children were not paying enough attention to the presented stimuli. Another limitation concerns sex balance across groups. Although we tried to include as many male participants in the control group as possible, the children with dyslexia and ISD had (insignificantly) more male participants. In consequence, it might be difficult to exclude possibility that sex had a certain effect on the differences between groups. It is known that there are more males with dyslexia diagnosis than females, this might be due to the greater variance of male performance in the low level of reading distribution (Arnett et al. 2017). Finally, to achieve whole brain coverage, we applied 4 mm slice thickness in contrast to standard 3 mm which might have led to a decrease in signal detection.

In summary, we have shown that the functional organization of the neural network for written word processing is altered differently in groups of children with deficits in reading and spelling. Both groups with dyslexia and isolated spelling deficit, with poorer phonological skills than typical readers and spellers, showed hypoactivations in the posterior superior temporal cortex in response to print and an altered pattern of the print–speech convergence in the language network. The left occipito-temporal cortex dysfunction in children with dyslexia seems to be independent from the phonological deficit (in agreement with Kronschnabel et al. 2013). This finding of hypoactivation was driven by an atypical pattern of higher activity to non-linguistic visual stimuli vs words, which indicates inefficient selectivity of the VWFA to words in children with dyslexia. In conclusion, the impact of reading and spelling deficits on word processing at the neural level is twofold. First, the atypical lexico-orthographic processing in the left vOT is associated mostly with the reading deficit. Second, the underspecified organization of the sublexical language network in the posterior superior temporal cortex is linked not only to poor reading, but also to poor spelling and possibly poor phonological skills.

## Supplementary Information

Below is the link to the electronic supplementary material.

Supplementary file1 (DOCX 20 KB)

## Data Availability

The data used for the current study are available from Open Science Framework: https://mfr.osf.io/render?url=https://osf.io/yns5p/?direct%26mode=render%26action=download%26mode=render. The link is currently available for peer-review and will be made public when accepted.

## Abbreviation

ISD: Isolated spelling deficit
