# Supplementary material for: Neural patterns of word processing differ in children with dyslexia and isolated spelling deficit
Source: Brain Struct Funct. 2021 Mar 23;226(5):1467–78. doi: 10.1007/s00429-021-02255-2 (PMC8096730; doi:10.1007/s00429-021-02255-2)
Supplement: Supplementary file 1 — Supplementary file1 (DOCX 20 KB) [file 429_2021_2255_MOESM1_ESM.docx]

Appendix S1.

Table S1. Significant brain regions activated in contrasts print> symbol strings, speech > vocoded speech and their overlaps across groups of children (ISD: Children with isolated spelling deficit).

|  | **Brain region** | **H** | **x** | **y** | **z** | **T** | **p** | **Voxels** |
| --- | --- | --- | --- | --- | --- | --- | --- | --- |
| **CONTROLS** | **PRINT** |  |  |  |  |  |  |  |
|  | Supplementary Motor Area | R, L | -4 | 4 | 62 | 5.92 | <0.001 | 1290 |
|  | Middle & Superior Temporal Gyri, Inferior Frontal (tri, orb, oper), Middle Frontal, Fusiform, Insula | L | -56 | -42 | 6 | 9.07 | <0.001 | 9636 |
|  | Thalamus, Caudate, Putamen, Pallidum | L | -16 | 2 | 6 | 5.06 | <0.001 | 1443 |
|  | Middle & Superior Temporal, SupraMarginal, Angular | R | 46 | -30 | 0 | 6.35 | <0.001 | 2771 |
|  | Inferior Frontal (tri, orb, oper), Insula | R | 60 | 30 | 20 | 4.14 | <0.05 | 617 |
|  | **SPEECH** |  |  |  |  |  |  |  |
|  | Middle & Superior Temporal, Inferior Frontal (tri, orb, oper), Rolandic Oper, Heschl | L | -58 | -4 | -6 | 15.9 | <0.001 | 8130 |
|  | Middle & Superior Temporal, Inferior Frontal (tri, orb, oper), Rolandic Oper, Heschl | R | 62 | -2 | -6 | 12.3 | <0.001 | 5936 |
|  | **OVERLAPS** |  |  |  |  |  |  |  |
|  | **Middle & Superior Temporal Gyri, Inferior Frontal (tri, orb, oper)** | **L** | **-52** | **-40** | **4** | **7.59** | **<0.001** | **3062** |
|  | **Middle & Superior Temporal Gyri** | **R** | **48** | **-34** | **-2** | **5.07** | **0.001** | **1328** |
| **DYSLEXIA** | **PRINT** |  |  |  |  |  |  |  |
|  | - |  |  |  |  |  |  |  |
|  | **SPEECH** |  |  |  |  |  |  |  |
|  | Middle & Superior Temporal, Inferior Frontal (tri, orb, oper), Rolandic Oper, Heschl, PostCentral, SupraMarginal | L | -60 | -8 | -2 | 14.4 | <0.001 | 9301 |
|  | Middle & Superior Temporal, Inferior Frontal (tri, orb, oper), Heschl | R | 58 | -24 | 2 | 10.8 | <0.001 | 5879 |
|  | **OVERLAPS** |  |  |  |  |  |  |  |
|  | - |  |  |  |  |  |  |  |
| **ISD** | **PRINT** |  |  |  |  |  |  |  |
|  | Precentral, Inferior Frontal (oper, tri) | L | -52 | 4 | 48 | 5.5 | <0.005 | 1208 |
|  | Inferior & Middle Temporal, Fusiform | L | -44 | -50 | -14 | 5.1 | <0.05 | 685 |
|  | Caudate | R,L | 14 | -6 | -8 | 4.3 | <0.01 | 916 |
|  | **SPEECH** |  |  |  |  |  |  |  |
|  | Middle & Superior Temporal, Inferior Frontal (orb), Rolandic Oper, Heschl | L | -60 | -14 | 0 | 11.2 | <0.001 | 4044 |
|  | Middle & Superior Temporal, Inferior Frontal (orb) | R | 62 | -22 | 0 | 9 | <0.001 | 2497 |
|  | **OVERLAPS** |  |  |  |  |  |  |  |
|  | **-** |  |  |  |  |  |  |  |

Note: height threshold p<0.005, FWEc, p<0.05.

Table S2. Significant brain regions activated in contrasts print> rest, speech > rest and their overlaps across groups of children (ISD: Children with isolated spelling deficit).

|  | **Brain region** | **H** | **x** | **y** | **z** | **T** | **p** | **Voxels** |
| --- | --- | --- | --- | --- | --- | --- | --- | --- |
| **CONTROLS** | **PRINT** |  |  |  |  |  |  |  |
|  | Inferior & Middle Occipital, Inferior & Middle Temporal, Fusiform, Cerebellum (crusl, VI), Lingual | L | -26 | -96 | - 6 | 10.7 | <0.001 | 7608 |
|  | Inferior & Middle Occipital, Fusiform, Cerebellum, Lingual, Calcarine | R | 38 | -90 | -4 | 9.62 | <0.001 | 4044 |
|  | Inferior Frontal (tri, orb, oper), Insula | L | -44 | 10 | 32 | 6.87 | <0.001 | 5686 |
|  | Middle & Superior Temporal, SupraMarginal | R | 62 | -48 | 10 | 6.65 | <0.01 | 933 |
|  | Inferior Frontal (oper, tri), Precentral | R | 58 | 30 | 28 | 5.54 | <0.001 | 2481 |
|  | Inferior & Superior Parietal Lobule, Angular | L | -30 | -64 | 46 | 5.2 | <0.001 | 1325 |
|  | Inferior & Superior Parietal Lobule, Angular, Precentral | R | 58 | -40 | 60 | 6.02 | <0.001 | 3055 |
|  | Supplementary Motor Area | L | -6 | 12 | 54 | 5.11 | <0.05 | 801 |
|  | **SPEECH** |  |  |  |  |  |  |  |
|  | Middle & Superior Temporal, Inferior Frontal (tri, oper, orb), Rolandic Oper, Heschl, Insula, PostCentral, SupraMarginal | L | -62 | -22 | 0 | 13.3 | <0.001 | 10884 |
|  | Middle & Superior Temporal, Inferior Frontal (tri, oper, orb), Rolandic Oper, Heschl, Insula, PostCentral, SupraMarginal | R | 60 | 0 | -10 | 12.1 | <0.001 | 10902 |
|  | **OVERLAPS** |  |  |  |  |  |  |  |
|  | **Middle & Superior Temporal Gyri** | **L** | **-54** | **-46** | **6** | **6.9** | **<0.01** | **1446** |
|  | **Inferior Frontal (tri, oper)** | **L** | **-44** | **18** | **24** | **4.5** | **<0.05** | **722** |
| **DYSLEXIA** | **PRINT** |  |  |  |  |  |  |  |
|  | Inferior & Middle Occipital, Inferior & Middle Temporal, Fusiform, Lingual | L | -26 | -96 | -12 | 8.8 | <0.001 | 3468 |
|  | Inferior & Middle Occipital, Inferior & Middle Temporal, Fusiform, Lingual, Angular, Calcarine | R | 34 | -92 | -4 | 7.8 | <0.001 | 4940 |
|  | Inferior Frontal (oper, tri), Precentral | L | -46 | 4 | 34 | 5.5 | <0.001 | 2301 |
|  | Angular, Inferior Parietal | R | 30 | -56 | 42 | 5.4 | <0.01 | 1132 |
|  | **SPEECH** |  |  |  |  |  |  |  |
|  | Middle & Superior Temporal, Inferior Frontal (tri, oper, orb), Rolandic Oper, Heschl, Insula, PostCentral, SupraMarginal, Angular | L | -62 | -26 | 8 | 16.8 | <0.001 | 12151 |
|  | Middle & Superior Temporal, Inferior Frontal (tri, oper, orb), Rolandic Oper, Heschl, Insula, PostCentral, SupraMarginal, Angular | R | 58 | -26 | 8 | 20.5 | <0.001 | 11202 |
|  | **OVERLAPS** |  |  |  |  |  |  |  |
|  | **Middle & Superior Temporal** | **R** | **50** | **-46** | **16** | **4.6** | **<0.05** | **897** |
|  | **Inferior Frontal (tri, oper)** | **L** | **-42** | **18** | **28** | **4.8** | **<0.05** | **1086** |
| **ISD** | **PRINT** |  |  |  |  |  |  |  |
|  | Inferior & Middle Occipital, Inferior & Middle Temporal, Fusiform, Cerebellum (crusl, VI), Lingual | L | -44 | -50 | -12 | 7.6 | <0.001 | 3621 |
|  | Inferior & Middle Occipital, Inferior & Middle Temporal | R | 32 | -98 | -8 | 7.6 | <0.001 | 1365 |
|  | Inferior Frontal (oper, tri), Precentral | L | -40 | -2 | 32 | 6.3 | <0.01 | 1095 |
|  | **SPEECH** |  |  |  |  |  |  |  |
|  | Middle & Superior Temporal, Rolandic Oper, Heschl, Insula, SupraMarginal, PostCentral | L | -62 | -20 | 8 | 12.6 | <0.001 | 7304 |
|  | Middle & Superior Temporal, Inferior Frontal (orb), Rolandic Oper, Heschl, Insula, PostCentral | R | 44 | -20 | 2 | 10.1 | <0.001 | 6799 |
|  | **OVERLAPS** |  |  |  |  |  |  |  |
|  | **-** |  |  |  |  |  |  |  |

Note: height threshold p<0.005, FWEc, p<0.05.
